# Antiulcer Effects of Methanol Extract of *Euphorbia hirta* and Honey Combination in Rats

**DOI:** 10.1155/2020/6827504

**Published:** 2020-11-20

**Authors:** Ifeanyi P. Onyeka, Sunday P. Bako, Mohammed M. Suleiman, Felix Afamefula Onyegbule, Ugonna C. Morikwe, Cyril Onyeka Ogbue

**Affiliations:** ^1^Department of Pharmacognosy and Traditional Medicine, Faculty of Pharmaceutical Sciences, Nnamdi Azikiwe University, Awka, Nigeria; ^2^Department of Veterinary Pharmacology and Toxicology, Faculty of Veterinary Medicine, Ahmadu Bello University, Zaria, Nigeria; ^3^Department of Biological Sciences, Faculty of Science, Ahmadu Bello University, Zaria, Nigeria; ^4^Department of Pharmaceutical and Medicinal Chemistry, Faculty of Pharmaceutical Sciences, Nnamdi Azikiwe University, Awka, Nigeria; ^5^Department of Pharmaceutical Microbiology and Biotechnology, Faculty of Pharmaceutical Sciences, Nnamdi Azikiwe University, Awka, Nigeria

## Abstract

Stomach ulcer is an endemic gastrointestinal disorder which constitutes a major public health problem all over the world. Stomach ulcer results when there is an imbalance between the protective factors (mucus and bicarbonate) and aggressive factors (acid and pepsin) in the stomach. Dried powdered leaves and stem of the phytomedicine *Euphorbia hirta* (*E. hirta*) (1000 g) was extracted with methanol using a soxhlet apparatus. The evaluation of the phytochemical constituents of *E. hirta* and acute toxicity (to ascertain the safety of using the phytomedicine over a short period of time) was carried out. The antiulcer and gastroprotective effects of crude extract of *E. hirta* combined with honey in rats were evaluated. The study model using 0.6 M HCl model of ulceration was used to evaluate the antiulcer and gastroprotective activities of the phytomedicine. The soxhlet extraction of *E. hirta* gave a yield of 54.5 g of crude extract (5.45%). Phytochemical screening of *E. hirta* showed that the extract contains alkaloids, tannins, saponins, glycosides, flavonoids, and unsaturated steroids. Acute toxicity studies showed that LD_50_ was greater than 5000 mg/kg. The study showed that the crude extract of *E. hirta* at 200 mg/kg when administered alone had 54% inhibition of ulceration while when administered together with honey increased to 94% inhibition of ulceration, but honey alone had 89.47% inhibition of ulceration. This implied that *E. hirta* when combined with honey had a synergistic effect and enhanced the inhibition of ulceration, and this could be seen by the protection of the gastric mucosa. The study of the phytomedicine *E. hirta* combined with honey revealed that the phytomedicine has antiulcer activities against 0.6 M HCl-induced gastric ulcer in rats. This therefore validates usage and claim by the Igbo people of the southeastern part of Nigeria that the phytomedicine of *E. hirta* combined with honey has good antiulcer potential.

## 1. Introduction

Stomach ulcer is an endemic gastrointestinal disorder which constitutes a major public health problem all over the world. Gastric ulcer occurs when there is a lack of balance between aggressive factors like the acid, pepsin, and local mucosal defense factors such as bicarbonate, mucus secretion, and synthesis of prostaglandins [1].

Gastric ulcer is characterized by the circumscription and complete loss of the gut epithelium in some parts of the digestive tract exposed to hypersecretion of hydrochloric acid and pepsin [2]. Treatment of ulcer has faced certain drawbacks such as unavailability of the drugs especially for rural dwellers, high cost of the conventional drugs, toxic effect of the conventional drugs, and ineffectiveness of the drugs.

Herbal medicine is a new area that could provide alternative for the treatment of the ulcer disorder. These herbal drugs are highly acceptable, accessible, cheap, readily available, highly potent, and relatively safe. In the southeastern part of Nigeria, the *Euphorbia hirta* (*E. hirta*) plant is called “iruru agwo” (i.e., snake weed) and is locally used for the treatment of snakebite and wounds. It belongs to the genus *Euphorbia*, family Euphorbiaceae. It is characterized by the presence of white milky latex [3]. It is usually erect, slender-stemmed, and spreads up to 80 cm tall (although sometimes it is seen lying down) [4]. It is an annual broad-leaved herb that has a hairy stem with many branches from the base to the top. The leaves are opposite, elliptical, oblong, or oblong-lanceolate, with a faintly tooted margin.

Studies had revealed that *E. hirta* has antibacterial [5], antihelmintic, antiretroviral [6], antiplasmodial, antiamoebic [7], antioxidant [8], sedative, antispasmodic, antifungal [9], and antimalaria [4] activities. The plant has been reported to contain alkaloids, triterpenoids, phytosterols, tannins, polyphenols, and flavonoids [3]. It is among the group of popular herbs used traditionally for the treatment of various diseases such as intestinal parasites, diarrhoea, peptic ulcers, heartburn, vomiting, amoebic dysentery, asthma, bronchitis, hay fever, laryngeal spasms, emphysema, coughs, colds, kidney stones, menstrual problems, sterility, and venereal diseases [4].

Honey, though it has been used as a vehicle in Ayurvedic medicine, has been reported to have antibiotic and wound healing effect [10] and also used for healing of cut and burns [11]. Also, the antimicrobial property of honey has been reported [12]. Honey has been viewed as a by-product of flower nectar and the upper aerodigestive tract of the honey bee; it is concentrated through a dehydration process inside the bee hive. The biological activity of honey is majorly determined by the biological sources of the nectar used by bees in the processing of honey. Plant-based drugs and other plant-based combination therapies have been viewed to be a potential source that is relatively clean and effectively safe as drugs, though not fully tapped. Mahmood et al. reported the antiulcer potential of honey combination with *Trigonella foenum-graecum* seed extract on experimental gastric ulcer in rats [13].

The choice of this present study was influenced by the folkloric claim by the Igbo people of Southeastern Nigeria that *E. hirta* combined with honey cures ulcer. Moreover, no scientific research work has been done concerning the antiulcer potential and the toxicity of the *E. hirta* combined with honey. The present study was undertaken to evaluate the acute toxicity, the preventive antiulcerogenic gastrotherapeutic potential of *E. hirta* combined with honey, and the phytochemical constituents of *E. hirta.*

## 2. Materials and Methods

### 2.1. Plant Material (Collection and Preparation)

Fresh whole plant (leaves and the stem) of *E. hirta* were collected from the main campus of Ahmadu Bello University, Zaria, Kaduna state, Nigeria. The plant was identified at the herbarium, Department of Biological Sciences, A.B.U. Zaria. A voucher specimen number 583 was deposited. The plant materials (leaf and stem) were air dried at room temperature 10-14 days during the dry weather condition. The air-dried plant was ground, and 1000 g of the powder was extracted using 5000 ml of 70% methanol by continuous extraction in a Soxhlet apparatus. The extract was concentrated to dryness. The concentrated extract was scrapped into a sample bottle and kept in desiccators until required.

### 2.2. Honey Collection

Honey used in the study was pure, unprocessed, unboiled, multifloral apiary honey from Apis mellifera Adansonni. The honey was collected from Forest Research Institute of Nigeria, Kaduna out-station, Kaduna state.

### 2.3. Animals

Healthy adult albino Wistar (63) rats (male) weighing between 150 and 170 were obtained from the Department of Veterinary Pharmacology and Toxicology, Ahmadu Bello University, Zaria. The animals were maintained at room temperature and humidity (25°C, 70% relative humidity) and allowed to acclimatize for 2 weeks. All the animals were fed with standard pelleted diet and water *ad libitum*. The study followed ethical guideline for investigations using experimental animals established by [14].

### 2.4. Other Materials

Cytotec® oral tablet manufactured by Pfizer Pharmaceuticals Ltd. was used. It contains 200 mcg of misoprostol, a synthetic prostaglandin E1 analog [15]. Misoprostol Cytotec is a water-soluble tablet. Pharmacodynamics of misoprostol has shown both antisecretory (inhibiting gastric acid secretion) and mucosal protective properties in animals [15]. Misoprostol can increase bicarbonate and mucus production [15]. Misoprostol was administered at 50 *μ*g/kg. The plant extract and honey combination was dissolved at 250 mg/ml of honey as stock concentrations.

Jenway spectrophotometer model 6405UV/VIS with serial number 3948, by Barloworld Scientific Limited (DUNMOW. ESSEX., CM63LB) was used.

### 2.5. Phytochemical Screening

Phytochemical analysis was conducted using the method described by [16] to determine the presence of secondary metabolites in *E. hirta*.

### 2.6. Acute Toxicity Study

Acute toxicity studies of the extract were made using the standard method of [17]slightly modified to ascertain the acute toxicity of the methanol extract of *E. hirta*. Briefly, nine animals (mice) were randomly allocated into 3 groups of 3 rats each. Animals in groups 1, 2, and 3 were given 10, 100, and 1000 mg/kg body weights, respectively, of the extract through the oral route. Animals were therefore monitored for signs of toxicity and mortality for 2 days (48 hours). Signs of toxicity and pathological findings observed were recorded appropriately. All the animals survived, so the extract was further subjected to acute toxicity test with higher doses in the second trial. In the second trial, 4 animals were randomly allocated to 4 groups of one animal each. Animals in groups 1, 2, 3, and 4 were given 1200, 1600, 2900, and 5000 mg/kg body weight, respectively, of the extract.

### 2.7. 0.6 M HCl Ulcer Model

Gastroprotective effect of crude methanol extract of *E. hirta* combined with honey was conducted as described by the method of [18]. Misoprostol was the standard drug used. Initially, the animals were fasted for 48 hours but were allowed free access to water *ad libitum*. They were randomly selected and divided into 9 groups of five rats each. Group 1 served as the negative control that received only water; group 2 served as the positive control that received misoprostol; groups 3 to 5 received the crude extract of *E. hirta* at doses of 200, 400, and 800 mg/kg body weight, respectively; groups 6 to 8 received a combination of 1 ml honey and crude extract of *E. hirta* at doses 200, 400, and 800 mg/kg combined with honey (1 ml), while group 9 received only 1 ml of honey. Thirty minutes after treatment, individual rats were given 0.6 M HCl (1 ml/rat) orally as an ulcerogen. Three hours after treatment with HCl, all the rats were sacrificed in a chloroform chamber. On each animal, ventral midline incision was made on the abdomen to expose the stomach. The ulcerated surfaces in each stomach were measured with transparent millimeter- (mm-) scale ruler, and the result for each group was expressed in mm of mean ulcer index [19].

### 2.8. Gastric Mucus

The concentration of gastric mucus was determined using the method of [20]. The excised glandular portion of the stomach (500 mg) was soaked in 0.1% *w*/*v* Alcian blue solution buffered with 0.05 M sodium acetate and HCl for 2 hours. The excess dye or the uncomplexed dye was removed by rinsing the stomach tissue twice in 0.25 M sucrose solution for 1 hour. The dye complexed with gastric wall mucus was extracted with 0.5 M MgCl_2_ for 2 hours. The extract was then shaken vigorously with an equal volume of diethyl ether, and the resulting blue emulsion was centrifuged at 5000 g for 10 minutes. The optical density of the solution was read against a buffer blank at 580 nm using a Jenway spectrophotometer, and the quantity of Alcian blue extract per gram wet stomach was then observed and noted.

### 2.9. Percentage Inhibition of Ulceration (PIU)

The percentage inhibition of ulceration was expressed as a percentage of the control by using the following formula as described by [21]:

(1) $$\begin{array}{c} \text{Inhibition}\text{percentage}\left(\%\right)=\left[\frac{{\text{UI}}_{\text{ulcer}\text{control}}-{\text{UI}}_{\text{treated}}}{{\text{UI}}_{\text{ulcer}\text{control}}}\right]\times 100. \end{array}$$

### 2.10. Statistical Analysis

All statistical analyses were done using Graphical Prism version 4.0 for Windows from GraphPad software, San Diego, USA. Data obtained were analysed using one-way analysis of variance. Where significant differences were observed, the Turkey post hoc test was used to identify and compare differences between groups. Values were considered significant if *P* < 0.05 and 0.01. Duncan multiple range test was used to compare the means across each treatment group with untreated group.

## 3. Results

One thousand grams (1000 g) of *E. hirta* gave an average yield of 54.5 g of the extract, and this gave a percentage yield of 5.45% when extracted with the continuous extraction process of the Soxhlet apparatus.

### 3.1. Phytochemical Screening

The result of the phytochemical screening is presented in Table 1. Phytochemical screenings of *E. hirta* showed that the extract contains alkaloids, flavonoids, tannins, saponins, glycosides, and unsaturated steroids while triterpenes and anthraquinones were absent.

### 3.2. Acute Toxicity Studies

The result of the acute toxicity studies is presented in Table 2. It showed that the LD_50_ of *E. hirta* is above 5000 mg/kg of crude extract because no death was recorded above 5000 mg/kg in the rats [22].

### 3.3. Ulcer Index

**Figure 1:**
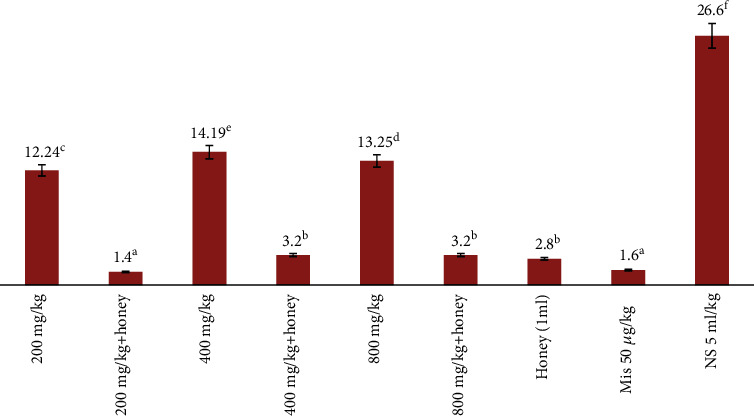
Effect of crude extract of *E. hirta* combined with honey on the ulcer index of rats using 0.6 M ulcer model. (NB: any two or more means having a common letter are not statistically different at the 5% level of significance.)

The result of the ulcer index of rats is presented in Table 3 and Figure 1. The result showed that the rats pretreated with misoprostol, honey alone, *E. hirta* alone, and in combination with honey at doses of 200, 400, and 800 mg/kg had a significantly reduced area of gastric ulcer formation compared to ulcer in the negative control group that received only normal saline. The result from examination of gross ulceration of the stomach showed that the negative control had the highest level of ulceration as indicated by the ulcer index followed by rats that received 400 mg/kg, 800 mg/kg, and 200 mg/kg of the crude extract of *E. hirta* respectively.

The result showed that rats that were pretreated with 200 mg/kg plus honey (1.4) combination had the least ulcer index followed by the rats that received misoprostol (standard drugs) (1.6), 400 (3.2), and 800 mg (3.2) of extract combined with honey when compared to the rats that were pretreated with 200, 400, and 800 mg/kg of extract alone and the negative control group.

The result showed significant reduction of ulceration (ulcer index) among rats treated with 200 mg/kg of the leaf extract of *M. oppositifolius* combined with honey when compared with all other treatment regimen. No significant difference was observed between the rat group treated with 800 mg plus honey and 400 mg plus honey compared with the group that received only honey (1 ml). Also, there was a statistical difference between the groups treated with honey (1 ml) and the group treated with the leaf extract at 200, 400, and 800 mg/kg alone. There was enhanced significant reduction in ulceration among the group treated with 200 mg+honey when compared with honey alone (1 ml) and when compared with 400 and 800 mg/kg plus honey. This implied that the crude leaf extract of *M. oppositifolius* at 200 mg+honey combined had enhanced significant therapeutic effect against ulceration in rats. This implied that the combination of honey and *E. hirta* crude leaf extract at 200 mg+honey showed significant synergistic effect in gross reduction of ulceration in 0.6 M hydrochloric acid-induced ulcers in rats.

### 3.4. Percentage Inhibition of Ulceration

**Figure 2:**
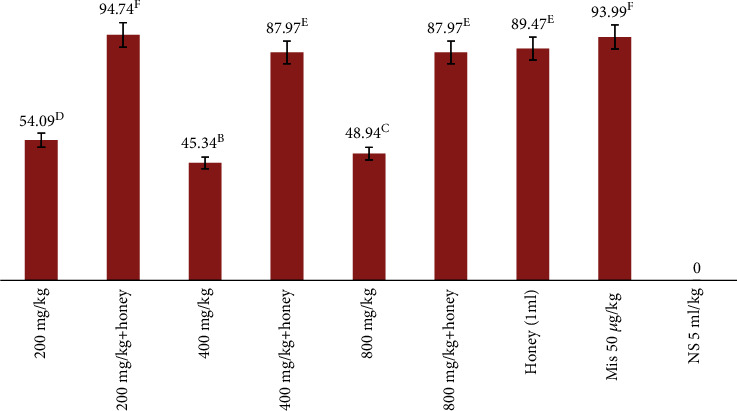
Percentage inhibition of ulceration of *E. hirta* combined with honey in 0.6 M HCl model ulceration in rats. (NB: any two or more means having a common letter are not statistically different at the 5% level of significance.)

The result of the percentage inhibition of ulceration is presented in Table 3 and Figure 2 (Table 3). The result of percentage inhibition of ulceration showed that rats pretreated with a combination of honey and *E. hirta* at 200 mg (94.74%) had the highest (significant) percentage inhibition of ulceration when compared with the rats pretreated with 200 (54.09%), 400 (45.34%), and 800 mg/kg (49.94%) of *E. hirta* alone and in 400 (88%) and 800 mg (88%) combinations with honey, respectively, while honey alone had 89.47% inhibition of ulceration, and this is significantly different. Also, the result clearly showed the synergy in percentage inhibition and antiulcer activity of the rats pretreated with *E. hirta* combination with honey when compared with those treated with only 200, 400, and 800 mg//kg of *E. hirta* extract alone. This implied that crude extract of *E. hirta* when combined with honey had better inhibition of ulceration when compared with the crude extracts that were administered alone to the rats. Although no significant difference was seen between the group that received honey alone (1 ml) when compared with the group that received *E. hirta* leaf extract at 400 and 800 mg/kg, respectively, combined with honey. There is significant difference between the group that received 200 mg *E. hirta* leaf extract plus honey when compared with the group that received honey alone (1 ml) and groups that received extract at 400 and 800 mg/kg plus honey, respectively. This implied that 200 mg/kg of the leaf extract of *E. hirta* plus honey worked synergistically to inhibit ulceration. There is no statistical difference between the group treated with 200 mg/kg of the crude extract plus honey when compared with the standard drugs; hence, they have similar therapeutic effect.

### 3.5. Stomach Weight

**Figure 3:**
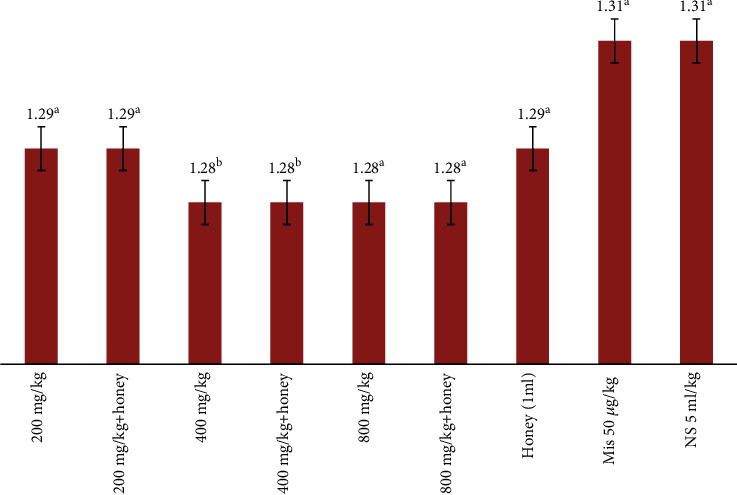
Effect of crude extract of *E. hirta* combined with honey on the stomach weight of rats using 0.6 M HCl (NB: any two or more means having a common letter are not statistically different at the 5% level of significance.)

The result of the effect of *E. hirta* when combined with honey on the stomach weight of rats is presented in Table 3 and Figure 3. The result showed that there was no significant difference in the stomach weight of rats among the entire treatment group. This implies that the weight of the stomach of the rats does not depend on the treatments. The result showed that the rats pretreated with 200 mg/kg and 200 mg/kg combined with honey had 1.29 g of the stomach weight while those that received 400 mg and 400 mg/kg combined with honey had 1.28 g of the stomach weight. The weight of the stomach of the rats pretreated with 800 mg/kg and 800 mg/kg combined with honey also was 1.28 g while honey alone had 1.29 g of stomach weight. The result further showed that misoprostol and distilled water had 1.31 g each of the stomach weight.

### 3.6. Gastric Mucus

**Figure 4:**
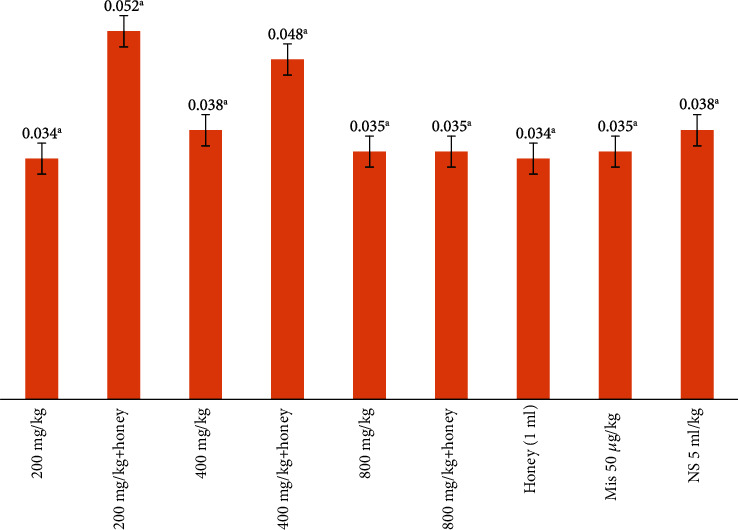
Effect of crude extract of *E. hirta* combined with honey on the stomach gastric mucus of rats using 0.6 M HCl.

The result of the effect of *E. hirta* on gastric mucus secretion on the stomach is presented in Table 3 and Figure 4. The study showed that the honey combined with *E. hirta* at 200 mg/kg enhanced the gastric mucus secretion, though there was no significant difference with the other treatment groups. Rats administered with 200 mg/kg combined with honey had the highest concentration of gastric mucus (0.052 *μ*g) while the rats administered with 200 mg/kg of the crude extract alone had the least 0.034 *μ*g of gastric mucus concentration. The result showed that rats administered with 200 mg alone had 0.034 *μ*g concentration of gastric mucus while the rat administered with 200 mg/kg combined with honey had 0.052034 *μ*g concentration of gastric mucus. This implied that honey combined with the crude extract of *E. hirta* had effect on the gastric mucus concentration which could be attributed to the cytoprotective nature of honey and the wound healing effect of honey which worked in synergy with crude extract of *E. hirta* to inhibit ulcers in rats.

## 4. Discussion

The present work evaluated the phytoconstituents, acute toxicity, and antiulcer effect of methanol extract of *E. hirta* combined with honey in rats. The result of phytochemical screening of *E. hirta* revealed the presence of alkaloids, tannins, saponins, glycosides, flavonoids, and unsaturated steroids. This is in agreement with the findings of [3, 23, 24] who reported similar phytoconstituents in *E. hirta*.

The result of the acute toxicity evaluation of *E. hirta* combined with honey showed no apparent toxic (mortality) effect in rats up to a dose of 5000 mg/kg when given orally. Therefore, LD_50_ of the extract combined with honey was considered to be above 5000 mg/kg of the extract [17]. This implies that the plant is a relative safe for consumption and is used in ethnomedicine, at doses not exceeding 5000 mg/kg of the extract.

Our choice of this combination therapy was informed by the traditional use of the leaves of *E. hirta* for the treatment of peptic ulcer in folk medicine and the well-established therapeutic values of honey, particularly in the treatment of gastrointestinal disorders. A combination therapy of the leaf extract and honey has an excellent antiulceration activity with a high potency.

The evaluation of antiulcerative effects of *E. hirta* combined with honey at doses of 200, 400, and 800 mg/kg using different ulcer models showed that the combination is effective. Gastric ulcer instillation using 0.6 mol/l HCl induces gastric necrotic damage due to infiltration of inflammatory cell leading to reduction in the secretion of bicarbonate, gastric mucus, and hypersecretion of nitric oxide. Instillation by 0.6 mol/l HCl reduces the gastric blood flow and induces the oxidative stress by increasing the production of malondialdehyde thereby reducing secretion of endogenous glutathione. The significant increase in the ulcer index gastric volume as observed in the negative group that received water after the instillation of ethanol confirmed the induction of ulcer, which can be attributed to either reactive oxygen species formation or inhibition of mucus synthesis and also lipid peroxidation. The gastric ulcer induced by 0.6 mol/l HCl could be associated with the increased purine degradation that leads to increased O_2_^−^ radical production and ROS-mediated increased lipid peroxidation. A low level of mucus suggests that the integrity of gastrointestinal apparatus was impaired when exposed to 0.6 mol/l HCl as seen in the negative control group. Treatment with the methanol leaf extract of *E. hirta* and the combination with honey showed that there was significant reduction in the ulcer index, most notably in the treatment with a combination of the extract and honey.

The antiulcer effect of *E. hirta* combined with honey could be attributed to antioxidant and free radical scavenging effect of the phytoconstituents of *E. hirta* and honey, respectively, as reported by several studies [25, 26]. Thus, the antioxidant effect of honey was due to its catalase content [25], and one molecule of catalase can scavenge for 40 million free radicals [26]. The antiulcer effect could have resulted from the cytoprotective effect of *E. hirta* combined with honey, which could be due to increase in mucus secretion that protects the gastric mucosal membrane from corrosive effects of HCl and stomach acid overproduction. This result is in agreement to the findings of [27] who reported that ethanolic extract *Euphorbia hirta* possesses gastroprotective potential which is related partly to preservation of gastric mucus secretion and antisecretory action. Also, the findings are similar to the findings of Mahmood et al. who reported that honey in combination with *Trigonella foenum-graecum* seed has antiulcer potential [13].

The combination of crude extract of *E hirta* and honey showed increased inhibition of ulceration, and this implied that there is synergy in activity. Flavonoids in *E. hirta* and catalase in honey by their capacity as a free radical scavenger [28] and saponins by their capacity to produce mucus could protect the gastric mucosal membrane against the acid effects and could synergistically be responsible for the antiulcer effect of these combinations.

Flavonoids enhance the protection of the gastric mucus therefore inhibiting ulceration. They stimulate prostaglandin bicarbonate and mucus secretion and prevent degrading effects of reactive oxidants in the gastrointestinal system [29]. Flavonoids have also been reported to offer protection in ulcer development by increasing capillary resistance and improving microcirculation [30]. It is acknowledged that tannins protect the outermost layer of the mucosa and make it less permeable and more resistant to chemicals and mechanical injury or irritation and thus prevent ulcer development [30]. Saponins induce mucus production, which protects the gastric mucosal membrane against the acid effects [31].

This finding further supports the veracity of the indigenous knowledge ethnomedicinal claim and has shown that *E. hirta* combined with honey is relatively safe for consumption since its LD_50_ exceeded 5000 mg/kg and also posses gastroprotective and gastrotherapeutic effect.

## 5. Conclusion

The result of this study suggests that the methanolic extract of *E. hirta* combined with honey is safe for use and could protect the gastric mucosa against damage by HCl and also has ulcer healing effect. The study confirmed that crude extract of *E. hirta* and standard drugs produced a decreased ulcer index and increased percentage inhibition of ulceration in 0.6 mol/l HCl-induced ulceration. Furthermore, the *E. hirta* crude leaf extract at 200 mg/kg combined with honey had more gastric mucus concentration which implied that the combination has an enhanced and synergistic effect against 0.6 mol/l HCl-induced ulceration in rats. The ulcer inhibition potential of *E. hirta* combined with honey could justify the use of this combination by the Igbo traditional healers in Southeastern Nigeria for the treatment of ulcer.

## Figures and Tables

**Table 1:** Phytoconstituents in the crude methanol extract of *Euphorbia hirta*.

Phytochemical compounds	Inference
Alkaloids	Present
Tannins	Present
Saponins	Present
Glycosides	Present
Flavonoids	Present
Anthraquinones/free antracene	Absent
Unsaturated steroids	Present
Triterpenes	Absent

**Table 2:** Acute toxicity test of methanol leaf extract of *E. hirta*.

Phase	Dose (mg/kg)	No. of animals	Death ratio
Phase 1	10	3	0/3
	100	3	0/3
	1000	3	0/3

Phase 2	1200	1	0/1
	1600	1	0/1
	2900	1	0/1
	5000	1	0/1

**Table 3:** Antiulcer effect of crude methanol extract of *E. hirta* combined with honey in rats.

Dosage	Ulcer index	Stomach weight	Gastric mucus	PIU
200 mg/kg	12.24^c^	1.29^a^	0.034	54.09^d^
400 mg/kg	14.19^e^	1.28^a^	0.038	45.34^b^
800 mg/kg	13.25^d^	1.28^a^	0.035	48.94^c^
200 mg/kg+honey	1.4^a^	1.29^a^	0.052	94.74^f^
400 mg/kg+honey	3.2^b^	1.28^a^	0.048	87.97^e^
800 mg/kg+honey	3.2^b^	1.28^a^	0.035	87.97^e^
Mis 50 *μ*g/kg	1.6^b^	1.31^b^	0.035	93.99^f^
Honey (1 ml)	2.8^b^	1.29^a^	0.034	89.47^e^
NS 5 ml/kg	26.6^f^	1.31^b^	0.038	—

NB: any two or more means having a common letter are not statistically different at the 5% level of significance.

## Data Availability

Data supporting the research findings are available in this manuscript, and any other information required shall be supplied based on request.
